# Clinical efficacy of urea treatment in syndrome of inappropriate antidiuretic hormone secretion

**DOI:** 10.1038/s41598-022-14387-4

**Published:** 2022-06-17

**Authors:** Eva Perelló-Camacho, Francisco J. Pomares-Gómez, Luis López-Penabad, Rosa María Mirete-López, María Rosa Pinedo-Esteban, José Ramón Domínguez-Escribano

**Affiliations:** Endocrinology and Nutrition Department, San Juan University Hospital, Alicante, Spain

**Keywords:** Endocrinology, Medical research, Nephrology

## Abstract

The aim of this work is to examine our experience in the use of urea in patients with SIADH. Observational retrospective analysis of 48 patients with SIADH that have been treated with urea in a third-level hospital. Pre-post analysis of serum sodium levels. The 48 patients with SIADH had a median age of 78.5 (range 26–97 years). The serum sodium nadir was 119.8 ± 5.0 mmoL/L and at the beginning of treatment 125.6 ± 4.1 mmoL/L. The patients continued the treatment for a mean time of 2.95 ± 6.29 months, being the treatment still active in 4 patients. In all patients there was an improvement in serum sodium, being the final serum sodium at the end of treatment 134.4 ± 4.9 mmoL/L (p < 0.01). This improvement was observed from the first week. Adverse events were only detected in 2 patients with mild digestive symptomatology and 2 patients refused the treatment due to the low palatability of the urea. There was an economic cost reduction of 87.9% in comparison with treatment with tolvaptan. Urea has shown to be a safe and cost-effective option for the treatment of hyponatremia caused by SIADH.

## Introduction

The syndrome of inappropriate antidiuretic hormone secretion (SIADH) is the clinical condition that results from the non-physiologic secretion of antidiuretic hormone, without taking into consideration changes in osmolarity or circulating volume, its normal stimulus. Consequently, there is an increased reabsorption of sodium-free water, that leads to an euvolemic hyponatremia. The SIADH is considered the most prevalent cause of hyponatremia, representing 30% of all the hyponatremia cases an up to 50% of all the chronic hyponatremia cases^1^. It is an exclusion diagnosis often done during the hospitalization process. Among the most common causes of SIADH are malignancy, pulmonary or neurological pathologies or pharmaceutical drugs^2–4^. Also in up to one third of the cases the etiology is unknown (idiopathic SIADH) and is frequent especially in older individuals^5^. It can also be caused by several symptoms that are common in hospitalized patients such as pain, nausea and vomiting. Depending on the etiology of SIADH the syndrome could be self-limited or chronic.

Hyponatremia has been associated in multiple studies to an increase in mortality and morbidity^6^, even chronic hyponatremia often considered as “asymptomatic” has been shown to be associated with concentration problems, memory loss and gait instability. This highlights the importance of a correct diagnosis and treatment^7^.

The treatment of hyponatremia caused by SIADH will depend on the length and severity of the symptoms. In the case of chronic hyponatremia, the treatment must be mainly etiological. If the underlying cause cannot be corrected, in the literature we can find several proposed strategies for its treatment. As a first-line treatment, the traditional option is fluid restriction, a medical strategy that is usually proposed as the first option in the main European and American clinical guidelines nowadays^2,8^. However, often due to specific patient characteristics, this common medical strategy is not possible to apply, or on the other hand, it is not very effective, sometimes because of the low therapeutic adherence. In consequence, we must resource to alternative pharmacological therapies, such as vaptans or urea.

There is certain controversy among professionals and medical centers over the most appropriate pharmacological treatment to apply. As a result, this has led to different approaches in the clinical practice^9^. In the current situation, vaptans or vasopressin receptor antagonists are the most frequently used therapy. In Europe, tolvaptan has been used since its approval in 2009, despite its high economic cost and its potential adverse effects like hyponatremia overcorrection and hepatotoxicity^1,10,11^. Risk of hepatotoxicity led the FDA to publish a safety alert in 2013 determining that it should not be used for longer than 30 days and recommending discontinuing tolvaptan in patients with symptoms of liver injury^12^. Demeclocycline and lithium, on the other hand, have been associated with nephrotoxicity and are rarely used nowadays^7^. The main American guidelines recommend urea, demeclocycline or vaptans as a second-line treatment, whereas European guidelines advise the use of urea or combined use of sodium chloride and loop diuretics^2,8^.

Urea behaves like an osmotic diuretic, being excreted in the urine after its administration. This produces an increase in the urine osmolality that leads to an increased excretion of sodium-free water, in order to achieve an elevation of serum sodium concentration improving the hyponatremia^13,14^. Urea is usually orally administered, although it could also be administered intravenously. The recommended dosage is generally 15–30 g/day and the maximum dose according to its drug data sheet is 45 g/day, although higher doses have been used sometimes in the intensive care units^15^. Previous research has confirmed the efficacy and safety of the urea in hyponatremia treatment^14,16,17^. This was first documented in 1980^18^ and, as we have already mentioned, its use is also recommended by the most important clinical practice guidelines. However, the use of urea in clinical practice is not widely extended maybe because of the lack of convincing evidences with limited and heterogeneous studies.

Considering all the above, the aim of this study is to examine the efficacy, efficiency and safety of urea for the treatment of hyponatremia caused by SIADH in a third-level hospital.

## Materials and methods

We performed an observational retrospective study of a case series of 48 patients with pre-post treatment analysis. We included 48 patients with a diagnosis of SIADH that had been treated with urea in our medical center, a third-level hospital. The study period was from November 2015 to April 2021 (a total of 5.5 years) and the sampling was done consecutively, including all the patients that met the inclusion criteria and received inpatient or outpatient care.

The selected patients met the following inclusion criteria: older than 18 years old, SIADH diagnosis of any etiology with symptom evolution of more than 48 h, and urea treatment prescription over the study period. The diagnosis of SIADH was made according to established international criteria, originally defined by Bartter and Schwartz in 1967 (Table 1)^2,8,19,20^. All the patients met the essential criteria; the supplementary criteria were not required to establish the diagnosis, but they were useful in cases of doubt. We defined hyponatremia as a serum sodium level lower than 135 mmoL/L. Accordingly to the severity, it was classified as mild (130–134 mmoL/L), moderate (125–129 mmoL/L) or severe (< 125 mmoL/L)^21^.

**Table 1:** Diagnostic criteria for SIADH.

**Essential criteria**
Clinical euvolemia: absence of signs of hypovolemia (orthostasis, tachycardia, decreased skin turgor, dry mucous membranes) or hypervolemia (subcutaneous edema, ascites)
Decreased measured serum osmolality (< 275 mosmol/kg)
Increased urinary sodium excretion (> 20–30 mmoL/L) with normal dietary salt and water intake
Inappropriate urinary osmolality (> 100 mosmol/kg)
Absence of other potential causes of euvolemic hypoosmolality: hypothyroidism or hypocortisolism, determined by clinical and laboratory assessment
No use of diuretic drugs
**Supplementary criteria**
Serum uric acid < 4 mg/dL
Blood urea nitrogen < 10 mg/dL
Fractional sodium excretion > 1%, fractional urea excretion > 55%
Failure to improve natremia after volume expansion with 0.9% saline infusion
Improvement of hyponatremia with fluid restriction
Abnormal water load test (excretion < 80% after 20 mL/kg water load in 4 h or failure to dilute urine > 100 mosmol/kg)
Serum AVP levels inappropriately elevated relative to serum osmolality

We included all the patients that met the inclusion criteria without any exclusion criteria. In the cases of severe hyponatremia, an infusion of hypertonic solution was used as the first treatment. The indication of urea was made by the physician responsible for the patient, according to our center protocol when it was considered that fluid restriction was not possible or effective. The treatment was supplied by the Hospital Pharmacy Service. Patients did not receive any concomitant drug for SIADH treatment other than urea and they did not fluid restriction during this period. Urea was dissolved in water, juices or other flavored liquids. The data collection was done retrospectively with a historical review of the urea prescriptions provided by the Hospital Pharmacy Service and an electronic clinical record review. The collected data were codified to warrant confidentiality.

For each patient, the collected variables were: age, sex, smoking habit, comorbidities (diabetes mellitus, dyslipidemia, arterial hypertension), SIADH etiology, urea dosage, total time of treatment and adverse drug effects. Biochemical variables were serum osmolality, urine osmolality, urine sodium levels, serum sodium nadir levels, serum sodium levels at the beginning of the treatment, during the treatment (at 7, 14, 30, 60, 120, 180 and 365 days), at the end of the treatment and 1 month after withdrawal of treatment.

The pre-post treatment analysis was executed using a t-Student test for paired data. The data were considered statistically significant if p < 0.05. Quantitative data are expressed as mean ± standard deviation. The statistical analysis was performed using the statistical software package Stata, version 13 (StataCorp, 2013; Stata Statistical Software; College Station, Texas, USA).

### Research involving human participants

This retrospective study was in accordance with the Helsinki Declaration. The study was approved by the Ethics Committee of the General University Hospital of Alicante (Spain), being this our reference accredited ethics comittee.

### Informed consent

The informed consent exemption was granted by the Ethics Comittee of the General University Hospital of Alicante (Spain) in view of the retrospective nature of the study, based on the law and the national ethical guidelines of our country^37^. All the procedures performed were part of the routine care. The collected data of the participating subjects were codified to warrant confidentiality.

## Results

We included 48 patients who met the inclusion criteria. In Table 2 we show their basal characteristics. The most frequent causes of SIADH were neoplasms (27.1%), drugs (16.7%) and idiopathic SIADH (14.6%). The serum sodium nadir was 119.8 ± 5.0 mmoL/L and at the beginning of urea treatment 125.6 ± 4.1 mmoL/L. None of the patients presented symptoms of hyponatremia at the baseline.

**Table 2:** Basal characteristics of the patients.

Variable	Participant subjects (n = 48)
**Sex**	
Male	50.0% (24)
Female	50.0% (24)
Age	78.5 (26–97)
**Smoker**	
Yes	22.9% (11)
No	77.1% (37)
**Comorbidities**	
Diabetes mellitus	39.6% (19)
Dyslipidemia	39.6% (19)
Hypertension	72.9% (35)
**Etiology SIADH**	
**Neoplasm**	27.1% (13)
Lung	16.7% (8)
Pancreas	4.2% (2)
Esophagus	2.1% (1)
Gallbladder	2.1% (1)
Brain	2.1% (1)
**Pharmacologic**	16.7% (8)
SSRIs	8.3% (4)
Carbamazepine	4.2% (2)
Opiates	2.1% (1)
Esomeprazol	2.1% (1)
Idiopatic	14.6% (7)
Pulmonary disease	12.5% (6)
Multifactorial	10.4% (5)
Neurologic	6.3% (3)
Others	12.5% (6)
Serum sodium nadir (mmoL/L)	119.8 ± 5.0
Serum sodium pretreatment (mmoL/L)	125.6 ± 4.1
Serum osmolality (mosmol/kg)	266.5 ± 14.8
Urine osmolality (mosmol/kg)	395.7 ± 128.5
Urine sodium (mmoL/L)	74.8 ± 39.1

Age is expressed as median (range). Other cuantitative variables are expressed as mean ± standard deviation. Cualitative variables are expressed as % (n).

*SSRIs* selective serotonin reuptake inhibitors.

The dose of urea used was variable among patients and was adjusted depending on evolution of serum sodium levels, according to the responsible physician criteria. The required doses were 15 g per day in 27.1% of patients, 15 g every 12 h in 50.0% and 15 g every 8 h in 22.9%. The total duration of treatment was 2.95 ± 6.29 months (range 1 day–14.9 months), being still active in 4 patients at the time of writing this manuscript.

**Figure 1:**
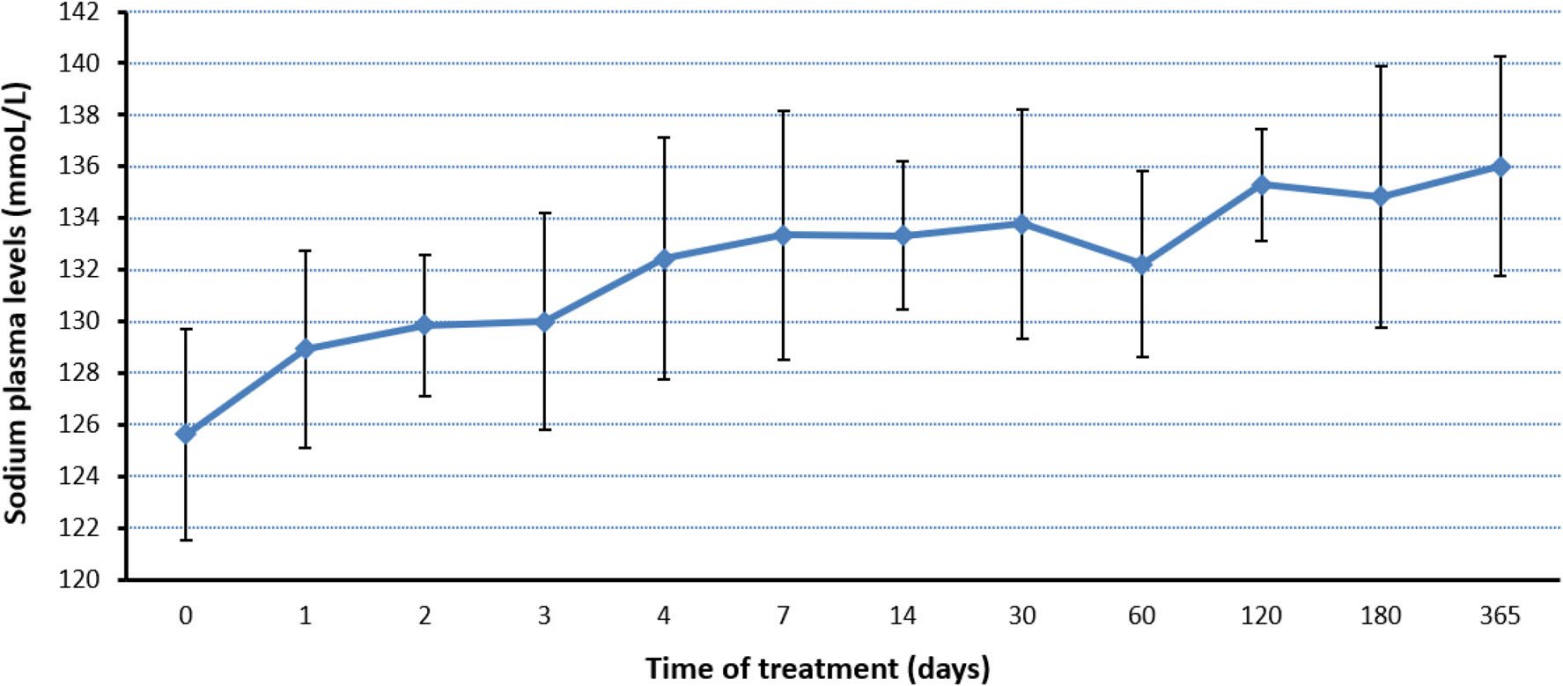
Evolution of sodium levels with urea treatment.

We observed an improvement of serum sodium levels in all patients with mean levels at the end of treatment of 134.4 ± 4.9 mmol/L, being this difference statistically significant compared to the initial sodium levels (p < 0.01). As a matter of fact, we found significant differences at the first week of treatment (p < 0.01), keeping sodium levels constant around 135 mmol/L during the total duration of the treatment. The evolution of serum sodium levels is exposed in Table 3 and graphically represented in Fig. 1.

**Table 3:** Evolution of plasma sodium levels with urea treatment.

Treatment time (days)	Sodium levels (mmoL/L)
0 (n = 48)	125.6 ± 4.1
7 (n = 35)	133.3 ± 4.9
14 (n = 18)	133.3 ± 3.0
30 (n = 14)	133.8 ± 4.6
60 (n = 9)	132.2 ± 3.8
120 (n = 7)	135.3 ± 2.4
180 (n = 6)	134.8 ± 5.6
365 (n = 4)	136.0 ± 4.9
End of treatment (n = 48)	134.4 ± 4.9

Sodium levels are expressed as mean ± standard deviation.

The treatment was interrupted by the patient in 4 cases (8.3%). The reasons of discontinuation were mild digestive symptomatology in two cases and limited palatability in other two cases. Two of them were shifted to tolvaptan and the others did not need any further treatment because of their positive evolution. There were no adverse events in the rest of the patients and none of them experienced an overcorrection of serum sodium levels (> 8–12 mmoL/L within the first 24 h of treatment). 9 patients died during treatment because of their underlying pathology and it was never related to the treatment. In the remaining patients, urea is still active (4 patients) or was stopped once a correct sodium level was reached, mostly when the cause of SIADH disappeared (31 patients). We did not have to discontinue the treatment because of the lack of efficacy in any patient. The serum sodium level one month after withdrawal of treatment was 137.1 ± 4.4 mmoL/L, confirming the correct SIADH resolution. There were no cases of lost to follow-up.

From the economic perspective, considering the mean duration of treatment (2.95 ± 6.29 months) and using the standard urea dose of 15 g every 12 h^5,22,23^ the total cost of treatment was 683.89 € per patient (9.42 €/day). Adding up all 48 patients the total cost of urea was 40,007.17€. If we compare this to the cost of tolvaptan during the same period and the same number of patients, using the Defined Daily Dose (DDD) of 30 mg/day (78 €/day), the total cost would have been 331,231.68€. This difference represents a total economic saving of 291,224.51€ with the use of urea as SIADH treatment (68.58 €/day/patient), that is equal to 87.9% of cost reduction. If we use the 15 mg dose of tolvaptan as comparison, the most widely used in the literature (and recommended in the official Prescribing Information as initial dose), the cost difference would have been the same, taking into consideration that the price of a 15 mg tablet is the same as the price of a 30 mg tablet.

## Discussion

Our study shows an increase in serum sodium concentration with the use of urea, in all patients and from the first week of treatment. This proves the efficacy of urea in patients with SIADH. Regarding safety, only two patients experienced mild digestive symptoms and there were no serious adverse events, showing evidence of the urea safety. It is true that the flavor of urea could influence a lower adherence to the treatment, but this only occurred in 2 patients. We agree with the literature that this problem has been traditionally magnified^8^. Furthermore, the flavor can be easily masked if urea is dissolved in orange juice or other flavored liquids, following administration recommendations. Although some studies have reported headache and hypokalemia as adverse events, we did not encounter any of those in our study^16^.

On the other hand, the data show the efficiency of urea due to the economic savings compared to the use of tolvaptan, a widely used treatment of SIADH, especially in long-term treatments. In 2020 a generic of tolvaptan was approved, but its price is also more expensive than the urea presentations available. If we also consider the potential adverse events of tolvaptan like the risk of overcorrection of natremia and the risk of hepatotoxicity, the use of urea is strengthened as a first-line cost-effective pharmacologic treatment.

The risk of overcorrection associated with tolvaptan has been related to lower initial serum sodium levels and lower blood urea nitrogen concentrations, as well as with oncological patients and those with low body mass index^24,25^. By contrast, this adverse event has rarely been described with urea and it has never been associated with osmotic demyelination syndrome^15,26^. As a matter of fact, studies in rats have even shown a protective role of urea against cerebral damage and osmotic demyelination syndrome^27^. In our study there were no cases of overcorrection.

Fluid restriction has proved to be effective in 59% of the patients with SIADH; possible predictors of lack of efficacy are urine sodium concentration > 130 mmoL/L and urine osmolality > 500 mOsm/kg^28^. This indicates that it is often necessary to use other pharmacologic strategies, when fluid restriction fails. Moreover, in some cases, adherence to fluid restriction is low or difficult to accomplish, as it happens in oncological patients. Oncological patients represent a large percentage of SIADH cases and many of them present a poor nutritional state or dehydration, so restricting their water access could be unsafe^17^.

We have not used fluid restriction concomitantly with urea, as some previous studies have done^29,30^, and even in this situation we have obtained good results. However, it would be recommended to do a comprehensive assessment of fluid intake in all patients. Some parameters have been proposed that indicate the need to use also fluid restriction to improve urea efficacy, such as urine volume and solute excretion^31,32^.

In agreement with previous studies, our study support the use of urea in the treatment of hyponatremia associated with SIADH^11,15,16,23,33^, which is also defended in the main recent clinical practice guidelines. There is evidence of its efficacy in the treatment of hyponatremia not only caused by SIADH but also due to heart failure, polydipsia and nephrogenic syndrome of inappropriate antidiuresis^34–36^. However, the use of urea is not yet widely extended.

We think urea should be considered the pharmacologic treatment of choice of chronic hyponatremia due to SIADH after ineffective fluid restriction or when this cannot be performed. Although this study does not directly compare urea to tolvaptan, for all the above we believe that vaptans should be considered a second-line treatment when urea is not well tolerated.

Limitations of our study include the retrospective nature of the data collection and the lack of a control group so that we can firmly obtain conclusive results. It would be important to design randomized controlled trials that allow confirming the therapeutic role of urea in the treatment of hyponatremia caused by SIADH. Nevertheless, the use of a case series is justified because it could exist an ethical conflict with the use of a control group, given the possible neurological adverse consequences of hyponatremia. We would propose a head-to-head clinical trial with tolvaptan, to find a higher level of scientific evidence. In our study, urea was used in real-life conditions, and the main measured variable (natremia) is an objective parameter, what helps to minimize placebo effect. To reduce the selection bias, we included all the patients consecutively. Besides, our study has a modest sample size, but it is the largest study in the literature of real-life treatment outside the intensive care units.

## Conclusions

According to our results, urea has proved to be a safe and cost-effective option in the treatment of hyponatremia caused by SIADH, showing improvement in serum sodium levels from the first week of treatment in all patients. For this reason, we believe that it should be considered a first-line pharmacologic therapy. However, these results need to be confirmed in randomized controlled clinical trials.

## Supplementary Information

Supplementary Information.

## Data Availability

All data analysed during this study are included in the Supplementary Information file S1.
