# Supplementary material for: Clinical efficacy of urea treatment in syndrome of inappropriate antidiuretic hormone secretion
Source: Sci Rep. 2022 Jun 17;12:10266. doi: 10.1038/s41598-022-14387-4 (PMC9206077; doi:10.1038/s41598-022-14387-4)
Supplement: Supplementary file 1 — Supplementary Information. [file 41598_2022_14387_MOESM1_ESM.pdf]

ID	Age (years)	Sex	Etiology SIADH	Na nadir (mmoL/L)	Na pretreatment (mmoL/L)	Na day 1 (mmoL/L)	Na day 7 (mmoL/L)
1	90	Female	Multifactorial	114	123		131
2	64	Male	Neurologic	117	124	130	130
3	81	Female	Multifactorial	117	125		
4	74	Male	Neoplasm	118	125	127	131
5	77	Male	Pharmacologic	122	129		142
6	82	Male	Pulmonar	123	125	133	132
7	97	Female	Multifactorial	117	129		
8	85	Male	Neoplasm	116	131	131	
9	71	Male	Pharmacologic	126	128	126	132
10	91	Female	Idiopatic	113	121	123	133
11	78	Male	Neoplasm	126	129		131
12	85	Male	Others	119	123		137
13	94	Female	Idiopatic	121	130		143
14	90	Female	Neoplasm	121	125		131
15	77	Male	Neoplasm	119	135		
16	83	Female	Pulmonar	119	124		132
17	74	Male	Neoplasm	128	128		138
18	77	Male	Idiopatic	107	116	125	
19	26	Male	Pharmacologic	124	126	130	132
20	61	Male	Neurologic	118	124	129	
21	58	Female	Neoplasm	111	127	130	131
22	85	Female	Neurologic	111	116	119	119
23	95	Male	Pulmonar	121	121		149
24	71	Female	Neoplasm	125	125	128	134
25	46	Female	Pulmonar	119	119	129	137
26	87	Male	Neoplasm	118	121	132	137
27	69	Male	Pulmonar	127	129		135
28	70	Male	Idiopatic	113	124	125	
29	29	Female	Others	118	123		
30	82	Female	Others	123	123	125	
31	82	Female	Pharmacologic	121	121	128	128
32	72	Male	Idiopatic	117	132	131	132
33	75	Female	Pharmacologic	121	128		132
34	93	Female	Others	127	127	129	132
35	90	Male	Idiopatic	123	123	129	131
36	79	Male	Pharmacologic	127	127	135	135
37	81	Male	Idiopatic	129	130		132
38	70	Female	Neoplasm	120	120	132	134
39	88	Female	Pharmacologic	124	125		128
40	82	Female	Neoplasm	125	125		135
41	58	Female	Neoplasm	113	134	138	132
42	89	Female	Pharmacologic	115	130		135
43	90	Female	Multifactorial	123	123	127	
44	82	Female	Multifactorial	118	127		
45	67	Male	Others	117	126		
46	66	Male	Others	120	130		131
47	77	Female	Pulmonar	114	125	128	
48	76	Male	Neoplasm	124	129	133	133

Na day 14 (mmoL/L)	Na day 30 (mmoL/L)	Na day 60 (mmoL/L)	Na day 120 (mmoL/L)	Na day 180 (mmoL/L)	Na day 365 (mmoL/L)
134	134			138	
130	128	134	136	127	130
137					
134	142				
130	130	124			
133					
134	131		139	143	142
138					
132					
	136	135	133	131	
	138	134			
129	136	136	136	136	136
	140				
132	129	134	137		
135	133	128	133		136
134					
133					
128	126	132	133	134	
137					
132					
	135	133			
138	135				

Na end of treatment (mmoL/L)	Na 30 days after withdrawal (mmoL/L)	Urea dose (g/day)	Duration of treatment (days)
138	138	30	176
130		15	29
127		15	448
137		15	28
142	145	30	38
139		15	39
131		15	88
128		30	76
133		30	7
142	142	30	353
138		15	35
132	144	15	20
137	137	15	245
135	140	30	84
135		15	21
132	134	30	7
142		30	8
133	138	45	3
133		30	979
140	140	30	23
137	137	30	125
147		30	723
149		45	5
128		30	18
137	136	45	4
137		45	7
135		15	8
125	141	30	2
134	138	30	5
129	133	30	5
128		45	1
133		30	36
134		30	293
132	127	45	8
131	131	45	3
135		45	2
132		30	4
136		45	9
128	129	45	2
135	136	15	15
137		30	16
135		30	13
134	136	30	2
129		15	0
132	139	30	3
132	138	30	57
133		15	107
135		45	67
